# Switching from Adalimumab Originator to Biosimilar in Patients with Hidradenitis Suppurativa Results in Losses of Response—Data from the German HS Registry HSBest

**DOI:** 10.3390/life12101518

**Published:** 2022-09-29

**Authors:** Natalia Kirsten, Frenz Ohm, Kathrin Gehrdau, Gefion Girbig, Brigitte Stephan, Nesrine Ben-Anaya, Andreas Pinter, Falk G. Bechara, Dagmar Presser, Christos C. Zouboulis, Matthias Augustin

**Affiliations:** 1 Institute for Health Services Research in Dermatology and Nursing (IVDP), University Medical Center Hamburg-Eppendorf (UKE), 20251 Hamburg, Germany; 2 European Hidradenitis Suppurativa Foundation e.V., 06847 Dessau, Germany; 3 Department of Dermatology, Venereology and Allergology, University Hospital Frankfurt, 60590 Frankfurt am Main, Germany; 4 Department of Dermatology, Venereology and Allergology, Ruhr-University Bochum, 44791 Bochum, Germany; 5 Department of Dermatology, Venerology and Allergology, University Hospital Wurzburg (UKW), 97080 Wurzburg, Germany; 6 Departments of Dermatology, Venereology, Allergology and Immunology, Dessau Medical Center, Brandenburg Medical School Theodor Fontane and Faculty of Health Sciences Brandenburg, 06847 Dessau, Germany

**Keywords:** hidradenitis suppurativa, biologics, TNF alpha, adverse drug reaction, biosimilar, drug effectiveness, switching, adalimumab, registry

## Abstract

Since 2021, adalimumab biosimilar ABP 501 can be used alternatively to adalimumab originator (ADAO) in the treatment of hidradenitis suppurativa (HS). Effectiveness and safety data remain scarce. We investigated the impact of switching from ADAO to ABP 501 on disease severity and the occurrence of adverse events (AEs) in patients with HS. We analyzed clinical data on patients enrolled in the German HSBest registry. Evaluation outcomes were assessed at three time points (baseline of originator (t0), prior to switching to biosimilar (t1) and 12 to 14 weeks after switching (t2)) and included patient-reported AEs and disease severity using the International Hidradenitis Suppurativa Severity Score System (IHS4) score. In total, 94 patients were switched from ADAO to ABP 501. Overall, 33.3% (*n* = 31/94) of the patients developed AEs and/or loss of response (LoR) within 12 to 14 weeks after switching. Of these, 61.3% (*n* = 19/31) experienced LoR but no AEs, 22.6% (*n* = 7/31) LoR combined with AEs and 16.1% (*n* = 5/31) AEs only. Our study showed that switching HS patients from ADAO to ABP 501 does significantly affect treatment effectiveness. Switching patients who are on remission maintenance therapy should be viewed critically.

## 1. Introduction

Hidradenitis suppurativa (HS) or acne inversa is a chronic, recurrent inflammatory skin disease. Typically starting after puberty, it manifests with inflamed lesions including painful inflammatory nodules, abscesses and non-/draining tunnels, most commonly located in axillary, inguinal, gluteal and perianal body regions [1,2]. Without adequate therapy, these symptoms may lead to scarring with functional limitation [3]. Treatment regimen depends on severity and includes topical and systemic antibiotics, surgery and biologics. In recent years, biologic medication has been used with increasing frequency in moderate-to-severe disease manifestation [4].

Adalimumab is an immunomodulating human monoclonal antibody inactivating tumor necrosis factor (TNF-) α [5]. This molecule appears to play a major role in pathogenesis of HS but also other diseases such as rheumatoid arthritis, psoriasis, psoriatic arthritis, Crohn’s disease and ulcerative colitis [5]. Clinical trials have demonstrated both high effectiveness and safety in the therapy of HS [6,7,8]. 

Since its approval by the U.S. Food and Drug Administration (FDA) in 2015, adalimumab is the first and still the only biologic approved for the treatment of hidradenitis suppurativa in Europe [9]. In most countries, the label includes an induction phase of 160 mg at baseline followed by 80 mg at week 2. Starting with week 4, either 40 mg weekly or 80 mg every other week are applied. 

Even though adalimumab has been a breakthrough in terms of quality of life improvement and clinical response rate, its use is associated with high costs. In response to this and in line with the originator’s patent expiration, increasing numbers of TNF-α inhibiting biosimilars from various pharmaceutical companies are emerging as cost-effective alternatives [10]. 

Biosimilars are biotherapeutic products structurally highly similar to the reference biologic and have been developed to show equivalent efficacy, quality, safety and activity but also are more accessible due to lower cost [11]. Unlike generics, which are identical copies of the original drug, biosimilars vary among themselves and from the originator. The reason can be found in the manufacturing process. In contrast to traditional drugs, biologics are manufactured from living cells. When working with living cells, the slightest differences in cell lines, materials used or laboratory conditions can significantly change the resulting medication [12]. Because of these inevitable differences in the manufacturing process, there will always be slight differences between a biosimilar and its respective originator [13]. If so, the prescription of a biosimilar instead of the originator could potentially be advantageous or disadvantageous for the patient due to higher or lower response rates and/or less/more adverse events (AEs).

We intended to obtain evidence on the clinical course in patients being switched from the adalimumab (ADA) originator (Humira^®^) to a biosimilar in patients with severe HS. The following research questions were addressed:

1. Does switching from adalimumab originator (ADAO) to biosimilar ABP 501 in HS patients have an influence on disease course?
2. What is the rate of non-response among patients with HS who were switched to biosimilar ABP 501?
3. Does disease severity have an impact on the response rate to ABP 501?
4. Do comorbidities affect the response to biosimilar ABP 501 in patients with HS?

## 2. Methods

### 2.1. Study Center and Data Collection

Adult patients with HS (≥18 years old) were prospectively enrolled in the German HS registry (HSBest). The data for the present study were collected at the Institute for Health Services Research in Dermatology and Nursing (IVDP) of the University Medical Center Hamburg-Eppendorf (UKE), which is a specialized center providing comprehensive guideline-based surgical and pharmacological care for patients with HS. 

For the current study, the following inclusion criteria were applied:

1. Treatment starts with ADAO after inclusion in the registry;
2. Treatment with ADAO between January 2021 and August 2021;
3. Treatment duration with ADAO of at least 24 weeks;
4. Hidradenitis Suppurativa Clinical Response (HiSCR) achievement prior to switching, defined as 50% reduction in total AN count, with no increase in abscess count, and no increase in draining fistula count relative to baseline [14].

Only fully documented datasets were considered for analysis.

Systemic routine therapy of HS was started with ADAO until January 2021. Following the drug regulation in Germany, all patients were then systematically switched to the adalimumab biosimilar ABP 501. 

The following data were collected: duration of therapy with ADAO prior to switching to ABP 501, disease severity at baseline of originator (t0) and severity and safety under originator at follow-up prior to switching to biosimilar (t1) and 12 to 14 weeks after switching to ABP 501 (t2).

Disease severity was determined by the number of inflammatory lesions such as nodules, abscesses and inflammatory tunnels. The International Hidradenitis Suppurativa Severity Score System (IHS4) score was calculated [15]. The Hurley Stage was also recorded at baseline before therapy with ADAO [16]. Safety data were obtained at each visit based on an explicit patient interrogation. 

### 2.2. Study Setting

**Figure 1:**
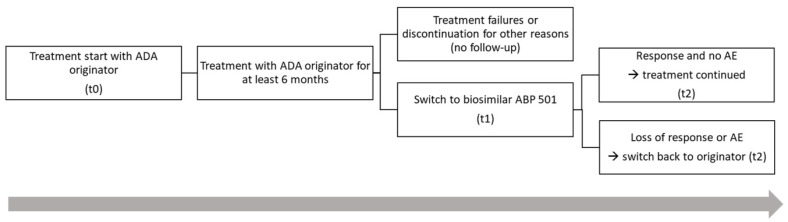
Distribution of 94 patients on systemic therapy according to the protocol (t0: baseline, start with adalimumab originator (ADAO); t1: switching point to adalimumab biosimilar (ABP 501); t2: outcomes measurement 12–14 weeks after switch to ABP 501). After induction (t0) and a treatment period of at least 6 months with originator ADAO, all patients were switched to ABP 501 (t1). At a follow-up 12–14 weeks after switch, response rate and side effects were captured.

Two groups were analyzed in comparison (Figure 1): The group of patients with good response and no AEs and the group of patients with a loss of response (LoR) and/or at least one AE. LoR was defined as an increase in IHS4 score of at least 50% compared to the switching point to biosimilar (t1). In a further step, the group of AEs was evaluated in more detail descriptively.

### 2.3. Statistical Analysis

Patient characteristics were first analyzed descriptively by using means, minimum, maximum and standard deviations. Non-parametric methods were applied to assess group differences and statistical significance; for metric variables, the Mann–Whitney U test was used and for ordinal variables the Pearson’s chi-squared or Wilcoxon test were used, where appropriate. Correlation analysis between variables and loss of effectiveness were performed using eta or phi coefficient as appropriate. Sign tests were also conducted for the IHS4 score, number of inflamed nodules, number of inflamed tunnels and number of abscesses. Comparison between patients with and without LoR was performed using Mann-Whitney U test, cross-tabulation, and Chi^2^ test. There were statistically significant results for the Hurley score and for IHS4 at t1 and t2. Statistical analyses were performed using SPSS 27.0.0 for Windows 10 (IBM, Armonk, NY, USA).

### 2.4. Ethics

Before the initiation of the registry, approval was obtained from the ethics commission of the State Medical Association Hamburg. Written informed consent of all patients was obtained before inclusion in the HSBest registry. The study was conducted in accordance with the Declaration of Helsinki.

## 3. Results

### 3.1. Demographic and Patient Characteristics

In total, 94 patients from a single HS center met the inclusion criteria and were selected for analysis. The mean age of the patients was 39.3 (±13.2) years, and 49% were male. The mean Hurley score was 2.26 (±0.7). Patients had a mean of 2.19 (±1.9) comorbidities and a mean of other pre-existing conditions of 0.4 (±0.7). They had received ADAO for a mean of 17.6 (±12.7) months before switching to ABP 501.

### 3.2. Descriptive Analysis of the Population with AE or LoR vs. the Non-AE/Non-LoR Population

Overall, 33.3% (*n* = 31) of the patients developed an AE or LoR within 12 to 14 weeks after switching from ADAO to ABP 501. Of these, 61.3 % (*n* = 19) experienced LoR but no AE, 22.6% (*n* = 7) LoR in combination with AEs and 16.1% (*n* = 5) AEs without LoR. The median duration of therapy under ADAO in this patient group (*n* = 31) was 19.0 (±13.3) months, without significant differences between patients with LoR and without LoR (Table 1).

**Figure 2:**
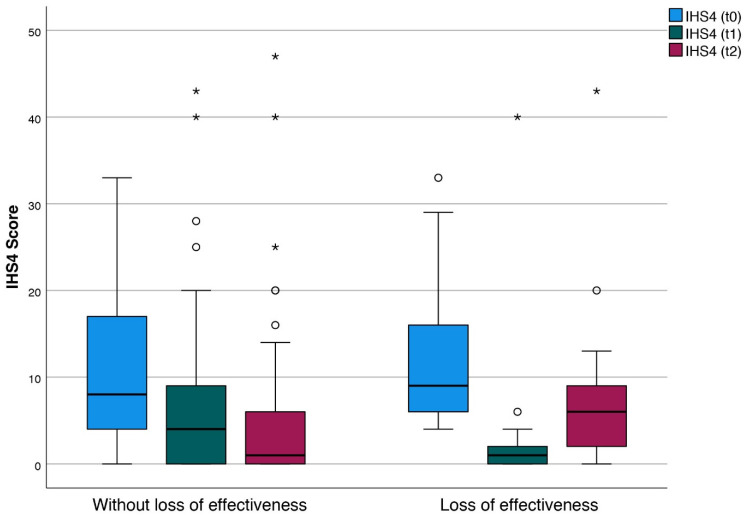
IHS4 course of patients (*n* = 89) on adalimumab treatment at 3 time points: before initiation of therapy with ADAO (t0), at switch to ABP 501 (t1) and 12 to 14 weeks after ABP 501 initiation (t2) depicting patients with loss of effectiveness (*n* = 26) versus patients keeping response (*n* = 63). ° 1,5-folt interquartile range. * 3-folt interquartile range. Abbreviations: IHS4 = International Hidradenitis Suppurativa Severity Score System, t = time point.

All the observed adverse events (*n* = 12) were mild and limited to the following symptoms: injection site pain (*n* = 6) and fatigue (*n* = 4) and pruritus (*n* = 2). The mean IHS4 scores at the start of ADAO treatment (t0) were comparable in the patients experiencing LoR (10.8 (±7.6)) and the patients without LoR (10.9 (±8.2)). At follow-up, mean IHS4 score was 2.4 (±7.2) and 6.6 (±8.9), respectively, at the time of switching to ABP 501 (t1), and IHS4 score rated 6.5 (±8.3) and 5.1 (±9.1), respectively, after 12 to 14 weeks of therapy with ABP 501 (t2) (Figure 2). 

### 3.3. Correlation Analysis between Variables and LoR

In the correlation analysis (Table 2), no correlation was observed between the effectiveness after switching and selected variables with regard to sex (*p* = 0.341), age (*p* = 0.367), number of comorbidities (*p* = 0.570), number of pre-existing conditions (*p* = 0.089), length of pretreatment with ADAO (*p* = 0.448), Hurley score (*p* = 0.262), number of inflammatory nodules (*p* = 0.474) or number of abscesses (*p* = 0.221). 

### 3.4. Statistical Analysis

Significant differences between t1 and t2 were identified for the IHS4 score (*p* < 0.001) in both patient groups and the number of inflammatory nodules and tunnels in patients with LoR (*p* < 0.05). 

## 4. Discussion

To our knowledge, this register-based monocentric study is the first to provide real-world data on the treatment of HS patients with the adalimumab biosimilar ABP 501 and on the process of switching from originator to this particular biosimilar during remission maintenance therapy. 

Our study results suggest that patients who underwent treatment switching to ABP 501 experienced a marked risk of LoR regarding an increase of tunnels after their reduction under ADAO and AEs. In the study population, 20.2% of patients experienced LoR after switching; 7.4% experienced LoR in combination with AEs; and 5.3% of patients experienced AEs without LoR.

A recent report by Montero-Vilchez et al. on switching from ADAO to an undefined adalimumab biosimilar in patients with HS comes to a comparable conclusion. In a relatively small cohort of 17 patients, a loss of HiSCR occurred in 5 patients (29.4%) after switching to the biosimilar. At the same time, 6 patients (35.3%) experienced side effects such as pain at the injection site, dizziness and nausea [17]. One clinical trial observed similar effectiveness and tolerability between originator adalimumab and biosimilar SB5 in 11 patients [18].

It cannot be excluded that the observed disease worsening in the HSBest cohort also would have occurred under continuation of ADAO therapy. However, a large proportion of secondary LoR in ADAO treatment usually occurs within the first year of application. By contrast, the observed LoR after switching to ABP 501 in our study occurred in patients who had already received ADAO for a mean of 19 months. Comparing our data with the data from the extension study of the PIONEER 1 and 2 phase 3 trials on the long-term efficacy of ADAO, it can be seen that secondary loss of efficacy in patients who have been assessed as partial responders at week 12 is about 28% over the three-year period. However, our patients have not received the treatment for this period of time. If we take week 72 as the point of comparison, about 6% of the patients suffered a loss of efficacy in the extension study. Looking at the efficacy curve of complete responders at week 12, the rate of secondary loss of efficacy is numerically 0% as measured by HiSCR. It should be noted, however, that our data are not one-to-one methodologically comparable with the PIONEER data [7].

Moreover, psychological aspects of switching to a “cheaper” reference product should not be neglected. Here, above all, the nocebo effect should be taken into account. Concerns about switching to a certain biosimilar include the possibility of safety issues, increased immunogenicity and LoR and may have influence the perception of treatment outcomes and AEs [19]. However, a nocebo effect would not influence the detected increase of tunnels under ABP 501 after initial reduction under ADAO. It is also possible that switching per se poses a risk to an LoR, regardless of whether it is from originator to biosimilar or vice versa.

On the other hand, the study results in the therapy of psoriasis, Crohn’s disease and rheumatoid arthritis indicate a good efficacy of ABP 501, so that in principle a comparable bioavailability and receptor affinity can be assumed in comparison to ADAO. In the studies conducted, however, the biosimilar was tested alone and not in comparison with ADAO. A study with ABP 501 in adalimumab-naive patients might have produced similarly good results in patients with HS. In order to generate valid results on the effectiveness and safety profile of biosimilars in the future, an active comparator arm with ADAO should be included in the study design to enable better comparability of the data.

In addition, one should bear in mind that there may also be indication-specific differences in response as well as in the safety profile. For this reason, it would be essential to take this into account in the approval process for medical products, especially biosimilars. Approval across indications without prior testing of effectiveness and safety for specific diagnoses is critical and requires further investigation. In addition, our study shows how important it is to generate real-world data [8,20]. Nevertheless, biosimilars in general provide an opportunity to increase patient access to biologic treatment. 

Some limitations should be considered when interpreting the observed study results. First and foremost, the short follow-up of 12 to 16 weeks should be mentioned. Further analysis will be needed to assess long-term effectiveness and safety. The performed open-label switch could have influenced the results, as a patient randomization would minimize the above-mentioned nocebo effect. Moreover, the study comprised a relatively low number of patients from a single German dermatologic outpatient center.

In conclusion, these results on 94 real-world patients with HS being switched from ADAO to ABP 501 permit the research hypothesis that ABP 501 is less effective and induces more AEs in a substantial number of patients with HS. Hence, the non-medical switch of patients to ABP 501—and potentially other biosimilars—who are on remission maintenance therapy with the ADAO poses risks. In the future, an unknown and unwanted substitution, performed by further treating doctors or pharmacists, could lead to a severe disease worsening. Instead, if an adalimumab biosimilar was selected for treatment of HS, it should be used at the very start of treatment, and multiple switches should be avoided. The option of starting with or switching to ADAO should further be considered. Further adjusted analyses from patient registries on HS with sufficient patient numbers are needed to further clarify the research hypothesis raised.

## Figures and Tables

**Table 1:** Comparison of disease course in patients with/without loss of response under therapy with ABP 501 at three time points: before initiation of therapy with ADAO (t0), at switch to ABP 501 (t1) and 12 to 14 weeks after ABP 501 treatment initiation (t2).

	Total Population (*n* = 94)		No Loss of Response (*n* = 63)		Loss of Response ± AE * (*n* = 31)		Differences between Groups
Variable	Mean	SD	Mean	SD	Mean	SD	*p*-value & Cohen’s d
Gender (1 = m; 0 = f)	0.49	0.50	0.52	0.50	0.42	0.50	0.385
Age	39.27	13.23	38.40	13.44	41.03	12.81	0.333
Hurley score (t0)	2.26	0.67	2.33	0.65	2.10	0.70	0.125
IHS4 (t0)	10.90	7.98	10.94	8.24	10.84	7.56	0.056
IHS4 (t1)	5.22	8.60	6.62	8.95	2.39	7.16	0.032, d = 0.47
IHS4 (t2)	5.57	8.80	5.14	9.06	6.45	8.31	0.007, d = 0.54
Number of comorbidities	2.19	1.89	2.27	1.91	2.03	1.87	0.553
Number of nodules (t0)	3.01	3.48	2.65	3.49	3.74	3.39	0.024, d = 0.23
Number of nodules (t1)	0.72	1.35	0.79	1.53	0.58	0.89	0.863
Number of nodules (t2)	0.99	1.94	0,56	1.53	1.87	2.38	<0.000, d = 0.43
Number of tunnels (t0)	1.83	1.88	1.94	1.98	1.61	1.65	0.605
Number of tunnels (t1)	1.12	2.06	1.43	2.12	0.48	1.81	0.001, d = 0.35
Number of tunnels (t2)	1.12	2.02	1.13	2.08	1.10	1.94	0.630
Number of abscesses (t0)	0.23	0.73	0.27	0.83	0.16	0.45	0.809
Number of abscesses (t1)	0.03	0.18	0.05	0.22	0.00	0.00	0.219
Number of abscesses (t2)	0.07	0.30	0.03	0.18	0.16	0.45	0.064

* under ABP 501 treatment SD = standard deviation, *n* = number of patients, m = male, f = female, IHS4 = International Hidradenitis Suppurativa Severity Score System, ADAO = adalimumab originator.

**Table 2:** Differences between responders and non-responders at different time points and correlation analysis between selected variables and loss of response.

Variable	Differences at t1 and t2 (*p*-Value)	Correlation between Variable and Loss of Efficacy (*p*-Value)
Age	-	0.367
ADAO in month	-	0.448
Sex	-	0.341
Hurley	-	0.262
IHS4	0.000 ^1^, 0.026 ^2^	0.237
Number of nodules	0.019 ^1^, 0.486 ^2^	0.474
Number of tunnels	0.000 ^1^, 0.109 ^2^	0.036
Number of abscesses	0.125 ^1^, 1.000 ^2^	0.221
Number of comorbidities	-	0.570
Number of pre-existing conditions	-	0.089

^1^ Loss of response; ^2^ Without loss of response; ADAO = adalimumab originator, IHS4 = International Hidradenitis Suppurativa Severity Score System.

## Data Availability

Data are available from the corresponding author upon reasonable request.
